# A Practical Guide to Participatory Design Sessions for the Development of Information Visualizations: Tutorial

**DOI:** 10.2196/64508

**Published:** 2024-12-13

**Authors:** Adriana Arcia, Samantha Stonbraker, Sabrina Mangal, Maichou Lor

**Affiliations:** 1 Hahn School of Nursing and Health Science, University of San Diego, 5998 Alcalá Park, San Diego, CA, 92110, United States, 1 619 260 7548; 2 College of Nursing, Anschutz Medical Campus, University of Colorado, Aurora, CO, United States; 3 School of Nursing, University of Washington, Seattle, WA, United States; 4 School of Nursing, University of Wisconsin-Madison, Madison, WI, United States

**Keywords:** audiovisual aids, cultural competency, health communication, patient participation, participatory design, information visualization, health literacy, user-centered design, human-computer interaction

## Abstract

Participatory design is an increasingly common informatics method to engage intended audiences in the development of health-related resources. Participatory design is particularly helpful for developing information visualizations that aim to improve health outcomes by means of improved comprehension, communication or engagement, and subsequent behavior changes. Existing literature on participatory design lacks the practical details that influence the success of the method and does not address emergent issues, such as strategies to enhance internet-based data collection. In this tutorial, our objective is to provide practical guidance on how to prepare for, conduct, and analyze participatory design sessions for information visualization. The primary audience for this tutorial is research teams, but this guide is relevant for organizations and other health professionals looking to design visualizations for their patient populations, as they can use this guide as a procedural manual. This start-to-finish guide provides information on how to prepare for design sessions by setting objectives and applying theoretical foundations, planning design sessions to match project goals, conducting design sessions in different formats with varying populations, and carrying out effective analysis. We also address how the methods in this guide can be implemented in the context of resource constraints. This tutorial contains a glossary of relevant terms, pros and cons of variations in the type of design session, an informed consent template, a preparation checklist, a sample design session guide and selection of useful design session prompts, and examples of how surveys can supplement the design process.

## Background and Significance

### Overview

Research from diverse fields (health, education, computer science, human-computer interaction, etc) has incorporated participatory design methods to ensure the products of research are acceptable to and effective among intended audiences [1-7]. Similarly, increasing interest in developing patient- and public-facing health visualizations has led to the uptake of participatory design methods within health informatics [8-11]. Furthermore, there is an existing and growing body of evidence indicating that well-designed visualizations can lead to better communication, heightened understanding of intended concepts, and other improvements in health outcomes, such as medication adherence when used in the health care space [12-14].

There are existing guidelines and recommendations on how to conduct participatory design sessions (the study by Spinuzzi [15] will be of greatest interest to readers) [1,15-17]. Some include its history and epistemological foundations [15]. However, we have learned that the efficiency, ease, and rigor of participatory design depend heavily on procedural details and practical considerations not addressed in existing guides, such as how to conduct internet-based sessions or track image iterations [1,15,18]. Therefore, this tutorial is in response to an acute need for a procedural and training manual specific to participatory design for information visualizations. We draw from our team’s collective experience designing health-related information visualizations through participatory design sessions with lay audiences and the current literature to provide detailed, practical guidance on conducting participatory design sessions.

### Definitions

Participatory design is a method for engaging members of the intended audience to develop a creative product, such as an information visualization [19]. It can ensure final products are culturally acceptable, visually appealing, and meaningful to intended audiences [7,20,21]. Within the larger sphere of user- or human-centered design methods, participatory design is one method that actively involves the intended audience [17]. If the intended audience consists of domain or visualization experts themselves, the activity may be more accurately described as a peer critique session and might best be served by different approaches (refer to the study by Semouchkina [22]). While both participatory design and focus groups gather feedback representing the group’s collective opinion [23], participatory design actively engages and works collaboratively with intended audiences in shaping how a visualization should look or function, whereas focus groups focus on exploring participants’ opinions or reactions to a topic without direct involvement in the design process. Textbox 1 presents a glossary of terms relevant to participatory design as used for the purposes of this guide.

Textbox 1. **Glossary of terms.**

- Participatory design: a method for collaborating with members of the intended audience to drive the development of a creative product, such as an information visualization. Often, it is an iterative process with changes made to the visualization between design sessions.
- Information visualization: a visual product, typically combining text and images, that has a communicative intent. Contrast with data visualizations, which are intended primarily for analysis and discovery.
- Design brief: a document defining the objectives, audience, content, and key requirements of a planned visualization.
- Graphical element: an image component, such as an icon or pictogram, of a larger visualization.
- Prototype design: a visualization that is still under development.
- Stimulus or stimuli: An umbrella term that includes graphical elements, prototype designs, and any other material presented to participants.
- Expert design phase: a collaboration during which the design team ideates and creates the initial graphical elements or prototype designs that will be presented in participatory design sessions. Design teams can include researchers, content experts, graphic designers, and illustrators.
- Graphic designer: a professional who uses text, typography, color, and images to create layouts, such as posters, pamphlets, etc.
- Illustrator: a professional who creates images via drawing, sketching, painting, etc.
- Generic infographic: an information visualization that has the same appearance for every viewer.
- Tailored infographic: an information visualization that varies in appearance because it incorporates data or information from or about the intended viewer.
- Design saturation: the point in the participatory design process at which participants express satisfaction with the stimuli and their feedback no longer leads to substantive design changes pertinent to the primary visualization objective. Put another way, the research team comes to consensus that they have arrived at the point of diminishing returns and concur that additional data will not further contribute to the accomplishment of the previously established visualization objectives.

**Figure 1.**
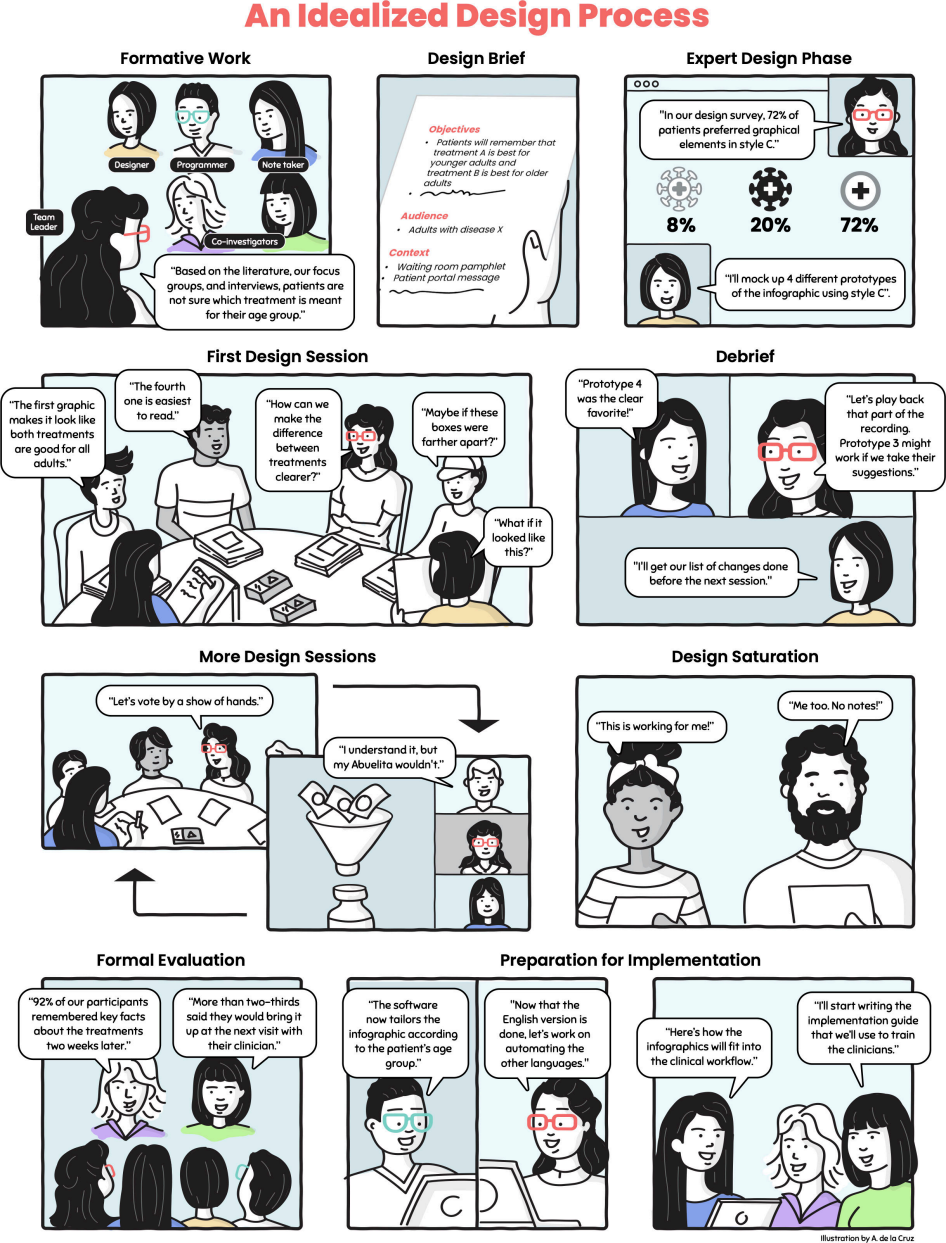
Design sessions are just one part of the larger visualization design process. This comic depicts an idealized design process to illustrate key concepts from this guide.

In this paper, we draw on our collective experience and current best practices in the literature to provide clear and detailed guidance on how to conduct participatory design sessions to create information visualizations. Multimedia Appendix 1 gives an overview of our team’s expertise and a table of case studies that summarize real-life scenarios of how 4 different studies implemented the recommendations offered in this guide, focusing on various populations and health issues. It is noteworthy that participatory design sessions are just one part of the broader visualization design process, as shown in Figure 1. Participatory design sessions are preceded by *formative work* (eg, literature review, interviews, and focus groups) that informs the *design brief*: a document defining the objectives, audience, content, and key requirements of the planned visualization [24,25]. We then engage a graphic designer or illustrator in the *expert design phase* to iteratively develop the prototype designs (stimuli) that kick off the *design sessions*. We find that offering participants some initial stimuli to respond to is more effective for actively engaging them in the design process than presenting them with a blank slate. We have previously described our process as a *hybrid* iterative participatory process to acknowledge the inclusion of the expert design phase in the process. We are discontinuing our use of the term *hybrid* to avoid potential confusion with meetings that involve both in-person and virtual participation.

Our methods were created and refined over time [26], align with best practices in participatory design (eg, meaningful iterative engagement with intended audiences), and are distinct from co-design approaches that incorporate participants from the earliest ideation phases and thus omit a phase that only involves experts [27,28]. Design sessions, carried out until reaching *design saturation*, can provide preliminary evidence that visualizations are meeting their objectives. However, *formal evaluations* (eg, comprehension testing, usability, and assessing behavioral intention or change) are essential for ensuring rigor and preparing for the later stages of the implementation phase, where their impact on health outcomes can be thoroughly evaluated. As one example, SS conducted a longitudinal assessment of the impact of an educational program using infographics for patients with HIV. The study found statistically significant improvements in HIV-related knowledge (proximal), self-efficacy to manage HIV (intermediate), and viral load (distal) outcomes [10,12]. In addition, Arcia et al [29] have formally evaluated infographic comprehensibility using the International Organization for Standardization (ISO) 9186 comprehension testing method, modified for interview-based (rather than written) administration [29]. *Preparation for implementation* includes activities such as software development [30], documentation, and training those who will deploy the visualization.

### The Structure of This Guide

We first discuss the importance of *setting visualization objectives* to define when visualizations are successful. Next, we address the importance of leveraging *theoretical foundations* for both the content domain and design decisions. We then summarize some of the variations we have applied to the *design session format* alongside their pros and cons. Then, the section on *preparation* covers practical concerns for preparing to conduct iterative participatory design sessions, such as ethical considerations, group composition, guidance on developing a robust design session guide, preparing stimuli for use in a session, and suggestions for preparing to track multiple iterations. Following that, there is a section on *conducting sessions*, where we discuss team roles, in-session tasks and prompts, navigating group dynamics, and procedures for concurrent data analysis and debriefing. The subsequent section focuses on what occurs *after sessions*, namely transcription, translation, and post hoc analysis. Next, we make suggestions about *working within resource constraints*, including highlighting options that can reduce costs. We close with a *discussion* of the value and limitations of participatory design and a brief *conclusion*. Readers are encouraged to refer to the Multimedia Appendix 1 for an overview of our expertise and case studies of 4 studies that used the methods in this guide, Multimedia Appendix 2 for an informed consent template, Multimedia Appendix 3 for a sample design session guide, Multimedia Appendix 4 for suggestions for supplementing design sessions with surveys, and Multimedia Appendix 5 for a preparation checklist that includes suggestions for the consent process, participant instructions, stimulus tracking, staffing, and supplies.

### Setting Visualization Objectives

We consider visualizations successful when they are culturally acceptable, visually appealing to the intended audience, and help the viewer achieve specific a priori objectives. Comprehension of personal health information has been the primary objective for much of our work based on the premise that comprehension is a necessary (although potentially not sufficient) precondition for impact on health outcomes. However, visualizations can serve other objectives, such as supporting clinical communication or emotional engagement [31,32]. For comprehension, the main discussion prompt might be “What do you think we are trying to tell you with this image?” whereas for engagement it might be “How does this image make you feel?” Consequently, it is important to have clarity and specificity about visualization objectives at the outset. Adar and Lee [33] provide excellent guidance on using the taxonomy proposed by Bloom to set visual learning objectives that are granular enough to be useful. For example, “understand the key differences between COVID-19 tests” is not as useful for supporting design decisions as “recognize that diagnostic tests detect current infection whereas antibody tests detect past infection.”

## Theoretical Foundations

### Overview

We use multiple theories to guide our work. Typically, one theory will be specific to the topic domain, and another will support design decisions. A deeper discussion of how specific theories can influence individual design decisions is beyond the scope of this guide but can be found in the study by Arcia et al [34].

### Content Domain

Theory selection should be based on the visualization objective and topic domain of the content. For example, if the objective is to encourage the viewer to take preventive health action, then the Health Belief Model suggests the included content should specifically address perceived susceptibility, severity, benefits, and barriers [35]. By contrast, if the objective is the promotion of physical activity, the choice of content could be informed by social cognitive theory and thus address questions of goal setting, self-monitoring, and feedback reinforcement [36,37].

### Design Decisions

To facilitate design decisions about how content should be visualized, we frequently rely on the Data-Frame Theory of Sensemaking proposed by Klein et al [38], and on the Conceptual Metaphor Theory proposed by Lakoff and Johnson [39,40]. The Data-Frame Theory of Sensemaking by Klein et al [38] suggests that people make sense of incoming data and stimuli by comparing them to the frames (schemas) that they have developed from prior lived experience. Consequently, sensemaking can be eased by using images and text that reference a frame that the viewer is likely to already have, such as stoplight colors to convey value judgments.

Conceptual Metaphor Theory treads similar territory in that it proposes that humans learn about new ideas (target domains) by drawing metaphorical comparisons to ideas that have become familiar through embodied experience (source domains). The logical consequences of the comparison are called entailments. For example, if arteries (target) are like plumbing (source), they can become clogged but also cleared. Parsons [40] points out that many graphical conventions feel intuitive not just because they are familiar but also because they use apt conceptual metaphors. For instance, in most charts, values increase upward along the y-axis rather than downward because our lived experience is that more is up. Applying this theory effectively means making design choices that deliberately evoke conceptual metaphors that have robust and accurate entailments. Design sessions are an opportunity to evaluate the extent to which design choices are functioning as intended and that unhelpful frames or conceptual metaphors are not being evoked unintentionally.

### Design Session Format Variations

Our initial design session format was to meet in person with a group of participants who had not previously seen any of the prototype visualizations. We have since varied aspects of the format as needed to accommodate the unique needs of each project, including budget and external circumstances (eg, virtual sessions due to pandemic lockdown). Within practical constraints, we choose a format—especially participant tasks—according to the likelihood that it will foster a successful design session by yielding actionable data. In Tables1  2, we describe format variations and summarize their pros and cons.

**Table 1.** Variations on participatory design session format and activities.

Variation	Good for	Cautions and caveats
**Number of participants per session**		
Individual: one participant takes part in the design session.	Eliciting granular feedback Stimuli with large amounts of content (eg, multipage documents) Design sessions held by videoconference	Generally requires more staff time per participant More difficult to discern when feedback is based on personal idiosyncrasy More sessions may be needed to reach design saturation than the group session format
Group: two or more participants take part in the design session.	Establishing consensus Stimulating discussion Efficient use of staff’s time Encouraging ideas or brainstorming	Comprehension assessment may not be robust because the first person to speak can influence others’ comments Can be hard to schedule, especially when grouping participants by shared characteristics. Need to manage group dynamics, such as one person dominating the discussion
**Venue**		
In-person: participants and research staff are physically present in the same room.	Observing body language, facial expressions, and other nonverbal cues Observing what areas of the page a participant is looking at (gaze following) Inclusion of people who lack devices, have a poor internet connection, and/or are not adept with technology Holding participants’ attention	Participants who are caregivers must arrange care for children or older people or be able to bring the care recipient with them Privacy and difficulty discussing sensitive topics
Virtual: a session is held via videoconference.	Minimizing travel time and geographic sampling restrictions Inclusion of people who are homebound, caregiving, or facing transportation difficulties Avoiding communicable disease transmission	Not recommended for groups because discussion is stilted and video and audio recordings may be of poor quality Technical problems, such as loss of internet connection, are common All parties must have a strong internet connection and be able to minimize background noise or distractions Participants must have comfort with the technology being used Harder for participants to sketch or indicate suggested changes during sessions Ability to evaluate body language may be limited
**Participant tasks**		
Elicit meaning: participants are asked to describe their interpretation of the stimulus.	Assessing comprehension and first impressions Exploring cultural associations	Individuals can only participate once because each must be naive to the stimuli being presented
Choose the best option: participants are told what the intended concept is (eg, depression) and asked to choose from among ≥2 stimuli (eg, graphical elements) the one that best represents that concept.	Narrowing down a pool of graphical elements Establishing consensus quickly	Not robust for assessing comprehension Researchers may need a second way to validate participants’ choices (eg, “explain your answer”), especially if participants are permitted to choose >1 option. Could limit participants’ creativity
Feedback: participants are asked for suggestions to improve the stimulus. They may also be asked to vote for their favorite(s) from among ≥2 stimulus options.	Generating actionable design changes Winnowing down a pool of prototype designs	Participants may make suggestions that violate basic design principles or are personal idiosyncrasies (eg, “I just don’t like the color blue”) Although directly contradictory feedback is possible, it is very rare
Generate new ideas: participants are encouraged to suggest design ideas beyond those already presented.	Expanding the scope of design concepts Exploring the mental models of participants	Participants may make suggestions that violate basic design principles; often they have no suggestions at all Lack of consensus on designs due to increased variety of ideas Many participants are not familiar with this type of task
Design surveys: printed or digital surveys can be used to collect data asynchronously for any of the above participant tasks.	Boosting the total number of participants and reaching consensus, especially when resources are limited Rapidly tallying preferences Informing design decisions before conducting design sessions	Survey data are not as rich and informative as data from design sessions Written responses are often terse and may not be interpretable; this limitation can be mitigated by allowing participants to audio-record their responses, but these must then be transcribed

**Table 2.** Variations on stimulus presentation and documentation.

Variation	Good for	Cautions and caveats
**Stimulus preview**		
No: participants do not see stimuli in advance of design sessions.	Assessing comprehension and first impressions (including nonverbal behaviors and interactions with the stimulus)	Must allow time for participants to read and review the stimuli; participants may be bored waiting for others
Yes: participants see the stimuli in advance of the design session. Options include sending printed material by mail or sharing files via email or SMS.	Stimuli with large amounts of content Thoughtful and considered feedback	May be logistically challenging to arrange In the interim, participants may forget some of the thoughts they intended to share Research staff relinquish control of the stimuli, which may be shared with others before designs are finalized Instructions in the stimulus must be very clear for participants to follow Participants might review the stimulus in a cursory manner or not at all
**Recording type**		
Audio only: only audio is recorded.	Easy and inexpensive documentation	It is advisable to use ≥2 recorders for adequate coverage if participants are seated far apart Recorders may run out of batteries or memory Transcribers must be able to distinguish between participants’ voices Participation is limited to those who consent to being recorded
Audio and video: both audio and video are recorded.	Design sessions held by videoconference Documenting nonverbal behaviors Matching the speaker to the voice	In-person design sessions may require multiple cameras and angles, including overhead, to provide useful levels of detail. Participants may be uncomfortable being video recorded Greater loss of confidentiality if unauthorized persons access recordings Participation is limited to those who consent to being recorded
**Note-taking**		
In a real-life situation: research staff take notes (free-form or in a template) during the design session.	Supporting rapid decision-making, identifying probing questions during a session, and facilitating analysis Supplementing transcripts and recordings	The notetaker may not be able to keep up with a fast-paced discussion or numerous participants
From a recording: research staff make notes (free-form or in a template) based on the review of a recording.	Thoughtful and thorough notes Reviewing moments of fast-paced discussion or when participants did not express themselves clearly Useful in the absence of a notetaker	Some initial observations and first impressions may be lost, especially if working only from an audio recording Can be time consuming

## Preparation

### Ethical Considerations

When used for research, participatory design requires institutional review board approval and an informed consent process. Multimedia Appendix 2 is a template that can be used for an informed consent form or for an information sheet if approval is obtained for waiving written documentation of consent. In most cases, the research will be considered minimal risk and will qualify for expedited approval.

### Group Composition

Participants must be grouped for sessions by language preference, including dialect (eg, Spanish speakers for Spanish-language visualizations). They may also be grouped by relevant characteristics, such as age, gender, or level of expertise (eg, newly diagnosed patient vs expert patient). Thoughtful groupings can be critical for some topics (eg, age and gender when discussing HIV) and of little importance for less sensitive ones. Occasionally, there is a benefit to deliberately mixing participants according to key characteristics. For example, while working with Hmong participants, ML has conducted sessions with mixed-gender groups, particularly including both husbands and wives who prefer to participate together. This gender composition aligns with the participants’ cultural values and fosters engagement. In practice, logistical constraints, such as recruiting on short notice, can limit the ability to maintain these groupings [41]. In these instances, it may be advisable to alert participants to potential discomforts (eg, a mixed-gender group) and remind them that participation is voluntary. Participants often benefit from multiple reminders (eg, date, time, and location) leading up to the session and should be reminded to bring their glasses, if applicable.

Occasionally, researchers have the luxury of selecting participants from a known cohort, such as individuals who have previously participated in studies with the same research team. In that case, the recruiter has the advantage of bypassing participants whose previous contributions were minimal in favor of those who not only articulate their opinion effectively but also reflect on how a stimulus influences their thoughts and emotions. Some participants struggle with this, especially if they have experienced few situations, such as higher education, in which they are regularly asked to engage in metacognition.

It is often important that participants be naive to the stimuli, particularly if the researchers wish to assess comprehension. If so, new participants must be recruited for each session. Otherwise, people may be invited to participate more than once.

### Design Session Duration, Size, and Number

In our experience, design sessions typically last 60 to 90 minutes, including consent and other paperwork (eg, demographic survey); sessions >120 minutes are inadvisable due to participant fatigue (people will just get bored and either leave or stop engaging). Previous research suggests piloting interviews to gauge the length of time that would be appropriate based on the intended audience [42]. We find a group size of 4 to 8 people to work well for most tasks, though we have run sessions with as few as 1 and as many as 15. Smaller groups (eg, 1‐3 people) are best if very granular feedback is needed so people do not get bored waiting to contribute. In addition, groups of >8 people can be difficult to manage, as it is rare for this many participants to wait for their turn to speak. The number of participants and whether individual or group sessions are needed will also depend on the team’s objectives or participant availability (eg, larger samples are harder to obtain with difficult-to-reach populations). In addition to providing more granular feedback, individual or smaller sessions are helpful for providing feedback on stimuli with large amounts of content and for virtual design sessions. However, they generally require more staff time per participant, may be difficult to discern feedback from personal biases, and may need more sessions to reach design saturation compared to group formats. Conversely, group formats may be useful when the objective involves establishing consensus on design decisions, stimulating discussion (eg, new ideas and brainstorming) between participants, and using staff time efficiently. However, within groups, participants’ opinions might influence others, scheduling conflicts might occur, and there is a greater need to manage group dynamics (eg, ensuring only one person does not dominate the discussion).

*Sessions should be continued until reaching design saturation*, which is when participants express satisfaction with the stimuli and their feedback no longer leads to substantive design changes pertinent to the primary visualization objective. Put another way, the research team comes to the consensus that they have arrived at the point of diminishing returns and concur that additional data will not further contribute to the accomplishment of the previously established visualization objectives.

It can be difficult to forecast how many sessions will be needed; we have done as few as 5 and as many as 21. The number of sessions needed for design saturation depends on group size (small groups may mean more sessions), the amount and complexity of the stimuli, how exploratory or novel the designs are, and the experience level of those making design decisions. Although the total number of participants and sessions matters, *the number of design iterations is of the greatest importance* because each iteration represents progress toward design saturation. The unpredictability of the design process makes it difficult to forecast how closely together sessions can be scheduled, especially at the beginning of the process, because the extent of the changes to be made before the next session is unknown. Occasionally, a freelance designer misses a deadline due to competing demands from other clients, which forces the research team to either postpone sessions or move forward without completing all of the planned changes to the stimuli. We suggest erring toward more, rather than less, time between sessions to avoid rushed work and rescheduled sessions.

### Venue and Environment

The ideal venue for sessions offers a quiet environment for clear recordings and is private, especially if discussing sensitive topics. In-person venues should be large enough so that all participants can see each other when seated or interact as needed. The lighting must be adequate for reading and not distort the colors in the stimuli. If sessions are only audio recorded, it is advisable to use ≥2 recorders for adequate coverage if participants are seated far apart and to have back-up power sources or batteries for recorders. In group sessions, researchers should keep in mind that they must be able to distinguish between participants’ voices when transcribing. While video recording in person, researchers should verify that camera angles and lighting provide detailed enough views of stimuli and participants’ reactions (eg, body language and facial expressions) to be useful. In-person venues can also be helpful for observing what a participant is looking at, actively engaging with participants, and providing accessibility for people who do not use or have access to the necessary technology for virtual sessions. However, maintaining privacy, potential discomfort with being video recorded, potential difficulty with discussing sensitive topics, accessing transportation, or arranging care for children or older adults might be challenges.

If the sessions are held via videoconference, team members should ensure that participants have comfort with the technology being used and have a strong internet connection as well as an environment where background noise or distractions can be minimized. It can be particularly challenging to maintain focus during the design session if participants join a video call from unsuitable environments, such as during a commute or in a loud and distracting area. It is also preferable if the participant’s full face is visible so the team can observe facial expressions and nonverbal cues. Furthermore, research teams can consider conducting training sessions to acquaint participants with the software before the design sessions [43]. The team must also be prepared with a plan for when participants encounter technical problems, such as loss of internet connection. Sessions via videoconference might become cumbersome with groups because discussion may be stilted, or audio may be of poor quality. However, videoconferencing is an effective method to facilitate video recording, minimize travel time and geographic restrictions for participant recruitment, and is particularly accessible for those who are homebound, caregiving, concerned about communicable disease transmission, or facing transportation difficulties.

### Design Session Guide

We have found that a good design session guide, similar to prompts for semistructured interviews, orients participants to the people present and their roles, expected duration, ground rules of interaction, the purpose of the study, funder, and activities planned (refer to the sample in Multimedia Appendix 3) [44]. To reduce socially desirable responses, we specifically request feedback about anything participants dislike or find confusing because otherwise, we cannot improve the visualizations. Examples of tasks and prompts to include in the guide depend on the goals of the project and can be found under the Conducting Sessions section.

### Stimuli

Our typical design process uses a “winnow and refine” approach (also known as “contracting the design space”) [17], where we start with as many options as possible and then use early sessions to winnow down to the most promising ones, which are then refined in later sessions (refer to the asthma infographic pedigree chart [page 34] of the paper by Arcia and Spiegel-Gotsch [45] for a visual example of this process). For simple visualizations, this often means preparing multiple variations on the same design concept (eg, vertical and horizontal format or different color palettes), each of which will be a unique stimulus. However, this approach can be impractical for visualizations that incorporate multiple graphical elements, so the initial stimuli can instead be variations on each element, such as 3 different versions of a hand-washing icon. Once the graphical elements have been winnowed down, they can be assembled into prototype designs that will each represent 1 stimulus. It can be hard for participants to imagine alternatives based only on description, so it is prudent to err on the side of preparing these alternatives in advance, including feedback and insight from participants where possible [17,46]. Another consideration for researchers is whether to provide a stimulus preview (ie, participants see the stimuli in advance of the design session). There are benefits to working with naive participants (ie, no stimulus preview), such as the ability to assess comprehension and first impressions, including nonverbal behaviors and interactions with the stimulus. However, researchers must allow time for participants to read and review the stimuli; participants may become bored waiting for others.

Researchers may choose to provide stimuli before the session through mail, email, or SMS text messages. This can be beneficial for getting feedback on stimuli with large amounts of content, and when thoughtful, detailed feedback is needed. However, the risks and challenges involved with this may include logistical challenges with coordination, a need for clear instructions for participants, loss of data if participants forget some of the thoughts they intended to share or neglect to review the material, and loss of control of the stimuli that may be shared with others before designs are finalized.

For in-person sessions, it is convenient for every person present to have their own printed set of all stimuli, in the planned order of presentation, that they can easily see and mark up directly. We prefer to print on cardstock as it takes less dexterity to handle than regular copy paper. As discussed subsequently, each stimulus should have a unique identifier on the back of the page or watermark in an inconspicuous footer. Sections of complex images can be numbered for easy reference. If printing is impractical, stimuli can be projected onto a large wall or screen.

For virtual sessions, stimuli can be shown via screen sharing. They can also be printed and mailed in advance with instructions to review in advance of the design session or not to open until the session, depending upon researcher preferences and objectives. Researchers should be aware that it may be logistically difficult to capture what participants sketch or annotate stimuli during sessions and should create a plan ahead of time to collect these data efficiently, whether that is through mailing in annotations, having an additional camera set up to show them, or annotating stimuli via screen sharing.

### Tracking Iterations

To facilitate analysis, it is very important to have a unique identifier for every iteration of a stimulus to enable tracking of even the smallest changes (eg, a typographical error). Numerous schemes are possible for assigning identifiers to stimuli and keeping track of them. The important part of tracking is that key metadata are captured for each iteration: the relationships between iterations, reasons for changes, and when or to whom iterations were shown. In a hypothetical example, participants in design session 1 (English, July 8) agreed that the font size was too small in C18a (parent iteration), so it was increased in C18b (child iteration) and shown to participants in design session 2 (Spanish; July 15). Previously, we used spreadsheets for tracking, but files can become large and unstable because of the inclusion of many images, even at low resolution. Some qualitative software packages can manage images and thus might be useful for tracking.

**Figure 2.**
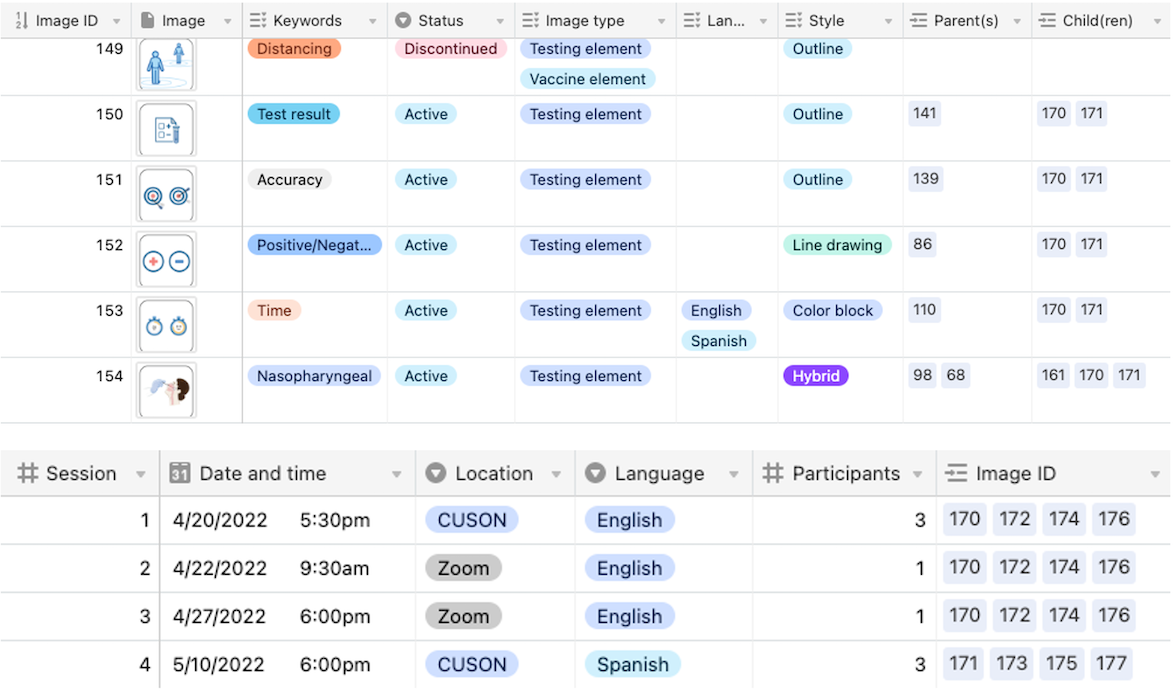
Excerpts from linked tables in a relational database. Top: each stimulus image is a record (row) and can be tagged with multiple attributes (eg, English and/or Spanish), linked to other records as a parent or child, and linked to specific design sessions (not shown). Bottom: each design session can be linked to the image IDs that were shown in that session.

To improve our process, we began using a commercially available relational database (Airtable [47]) that allows image files of stimuli to be dropped into individual records (rows) as shown in Figure 2. Records can then be tagged with multiple attributes and linked to one another as a parent or child. We use additional columns (not shown) for action items (ie, a summary of proposed design changes), completed actions, and notes. These records can also be linked to another table that lists the design sessions and their attributes (eg, date, time, location, language, number of participants, and stimuli shown) and, if needed, to files and artifacts containing details of complex design changes. A good tracking system facilitates summarizing what occurred throughout the design sessions, including how many stimuli were shown in a session and the total number of participants who saw any individual stimulus. If the design process itself will be analyzed for transferable insights, tracking—the centerpiece of the audit trail—is essential for qualitative rigor. Tracking has less importance if the design process will not be analyzed.

### Structured Note Templates

**Figure 3.**
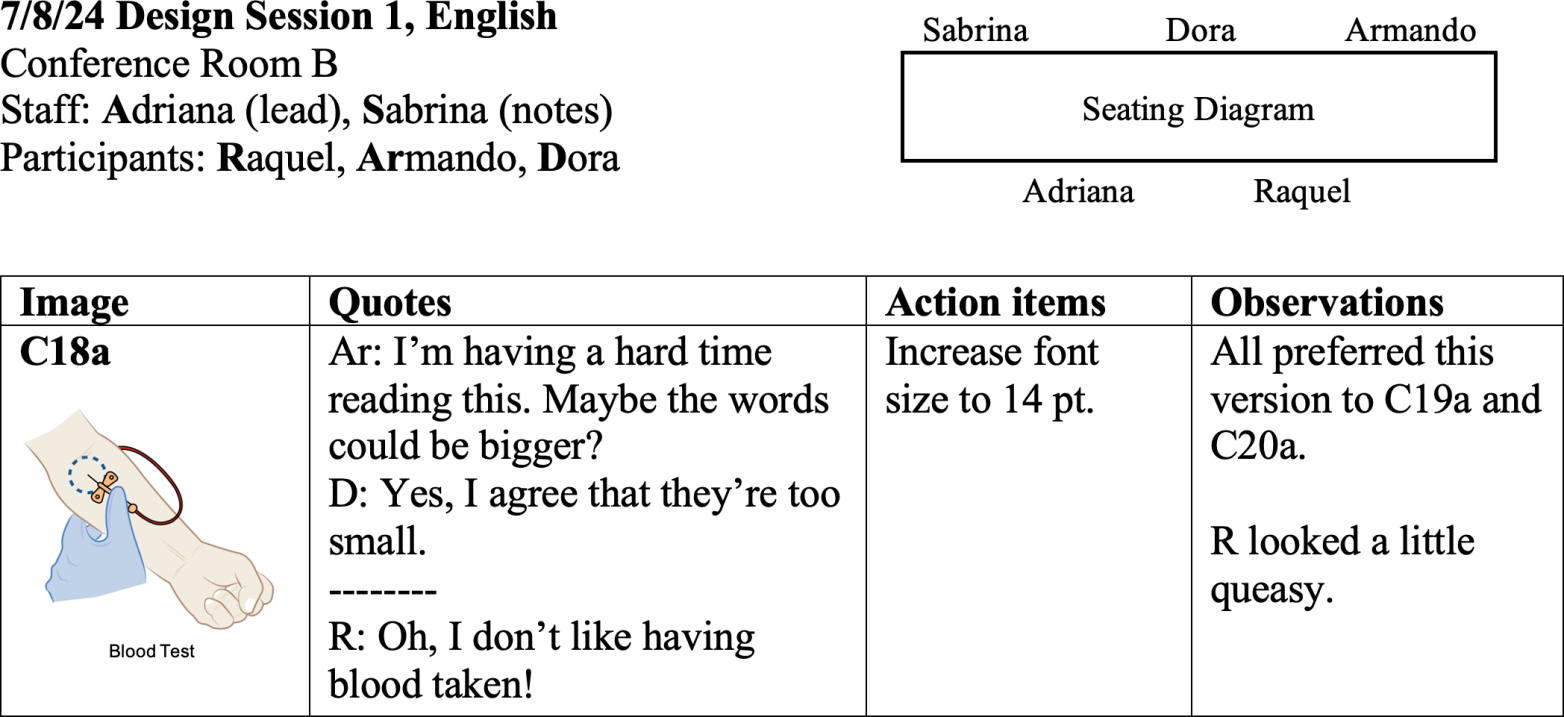
Excerpt of notes made using a template. Each stimulus has its own row and is shown in a thumbnail image in the first column, labeled with a unique identifier. The second column is for direct quotes from participants relating to the stimulus. Action items in the third column are planned tasks to be carried out in a subsequent iteration. The last column is for the notetaker’s observations, including body language, discussion summaries, and commentary on direct quotes, including emerging themes.

A structured note template helps to support both note-taking and analysis. In the hypothetical example in Figure 3, there are columns for stimulus ID codes and thumbnails, direct quotes, action items, and observations. Thumbnail images of stimuli are prepopulated in the left-hand column so the staff can quickly jot down an action item or make a note alongside its corresponding stimulus during sessions. Note-taking during a design session can support tracking rapid decision-making, help identify probing questions during a session, and facilitate analysis. These notes can serve as a supplement to transcripts and recordings.

Issues raised when the research team debriefs after a session are summarized in bullet points at the end of the document. After the session, direct quotes can be pulled from the transcript to substantiate participant perceptions, researcher observations, and action items for design changes. The criterion for what to include in notes (ie, “anything interesting”) is deliberately broad because even tangential or seemingly off-topic observations can become useful in aggregate during later analysis. Research staff may also make notes based on a review of a recording. This stage of note-taking can be helpful for thoroughly reviewing moments when participants did not express themselves clearly or the discussion was very fast paced and can also be helpful if no notetaker was present during the design session. However, if this is the only form of note-taking, it can be time consuming, and some initial observations and impressions about phenomena, such as body language, may be lost, especially if only working from audio recordings.

## Conducting Sessions

### Team Roles, Responsibilities, and Competencies

Ideally, the session leader, who is typically the head of the research or design team, should be trained and have experience with qualitative data collection techniques. Furthermore, the session leader should be very close to the project, with a clear understanding of the objectives of the visualizations and overall project goals. During a session, the session leader guides the discussion and is ultimately responsible for design decisions and content accuracy. In our experience, if the session leader is not part of the core design team, the data they elicit and their follow-up probes do not fully satisfy the questions that arise in the design process. Standard qualitative rigor techniques (eg, bracketing) serve to minimize bias [48]. It is helpful in each session to have at least 1 notetaker. Ideally, the notetaker is experienced with qualitative methods but, at a minimum, has been trained on how to take notes on the provided template. The more comfortable the notetaker is with the provided stimuli and project goals, the better [25]. The notetaker’s responsibilities are to keep track of participants’ reactions to the stimulus and nonverbal cues during the design session and conduct a review of the recordings. Other team roles may include project management (eg, scheduling participants and supporting videoconference sessions) and a designer or illustrator. If the latter cannot be present, a team member must clearly and promptly communicate design decisions in a way that is actionable for the designer. Depending on expertise, team members may take on more than one role. All team members who interact with participants should complete safety of human participants training before beginning study activities. For more guidance on working with the designer or illustrator, please refer to our team’s related publication [25].

### Tasks and Prompts

Upon starting the design session recording, have everyone present state their name or pseudonym so that voices can be properly attributed during subsequent transcription. It is important to ensure that everyone is looking at the same stimulus when they are providing feedback. Contextualize each stimulus by explaining what it is (eg, pamphlet or app screen) and when or where the viewer would encounter it (eg, “your nurse would discuss this with you during a visit”). The instructions and open-ended prompts relate to the objective of the visualization and the task at hand. Descriptions and examples of participant tasks are given subsequently.

*Eliciting meaning* or asking participants to describe their interpretation of a stimulus can be used to assess comprehension and first impressions or explore cultural associations. However, participants can only participate in the design process once because they must be naive to the stimuli being presented. The example prompt is as follows: “For this next page, please tell me what you think we are trying to tell you with this information, even if it seems really obvious.” Additional probing is often needed to reveal problem areas. For example, the leader may say, “Talk me through what you think is happening in the middle section,” and the participant may state that one should mix soap with hand sanitizer or realize, “Actually, this part isn’t very clear.” When showing multiple stimuli on the same topic, consider starting with the one expected to be most challenging to comprehend because otherwise, participants will learn from “easier” designs.

*Choosing the best option among graphical elements* can be applied by first telling the participant what the intended concept is (eg, depression) and asking to choose from among ≥2 stimuli that best represent that concept. This task is helpful when narrowing down a pool of graphical elements or establishing consensus quickly. However, it is not acceptable for assessing comprehension, and researchers may need a second way to validate participants’ choices (eg, “Explain your answer”), which may limit participants’ creativity. This task is distinct from *eliciting meaning*—usually, one or the other is selected based on the objective of the design sessions. The example prompt is as follows: “We are trying to get across the idea that someone needs help walking. Do any of these icons say that clearly? Which one(s)?”

*Feedback* can be used to elicit suggestions to improve the stimulus or as a way for participants to vote for their favorite(s) from various stimulus options. Feedback is helpful for generating actionable design changes and winnowing down a pool of prototype designs. Researchers should be aware that participants may make suggestions that violate basic design principles, reflect personal idiosyncrasies (eg, “I just don’t like blue”), or provide contradictory feedback. The example prompt is as follows: “How can we make this easier to understand?”

*Generating new ideas* can be used for participants to suggest design ideas beyond those already presented. This task is helpful for expanding the scope of design concepts or exploring the mental models of participants. However, similar to *feedback*, participants may make suggestions that violate basic design principles, may not have any suggestions at all, or may not be familiar with this type of task. There may be a lack of consensus on designs due to the increased variety of ideas. the example prompt is as follows: “What other images would help tell the story?”

*Design surveys* can be print or digital surveys used to collect data asynchronously for any of the aforementioned participant tasks. They can be useful for choosing design elements, boosting participant numbers to reach consensus, rapidly tallying preferences, and informing design decisions before design sessions (Multimedia Appendix 4). However, survey data are not as rich and informative as data from design sessions, and written responses are often terse and may not be easily interpretable.

Staff should reorient participants whenever the task changes and provide sufficient context so participants are clear on what the task is. The leader should pay particular attention to comments that reveal the participants’ mental models (eg, “bad scores should be on the right because dementia only gets worse over time”) [49]. Before closing, ask participants (1) what was missing from the content they saw, (2) if they have any unanswered questions, and (3) if there were questions that they should have been asked but were not. Close by thanking participants for their valuable contributions and reminding them to keep the conversation confidential.

### Preserving Respect and Dignity

It is of utmost importance that participants feel that their contributions are appreciated even if their comments are surprising, tangential, factually inaccurate, or strange, as sometimes happens. This may be especially true when participants have low general, or health, literacy. In these situations, it is important to maintain an unflappable demeanor (ie, a good “poker face”) and respond with openness and curiosity (eg, “That perspective is new to me! What are you looking at that led you to that conclusion?”). It can also mean providing a face-saving cover when eliciting comments that might reveal a lack of comprehension or other stigmatizing situations (eg, “Is there anything here that *other people* in your community might find hard to understand?” “Maybe someone you know has problems with alcohol. What might they think about this image?”). Furthermore, if the session leader notices that a participant has lower literacy or seems to be struggling with reading, they can thoughtfully avoid putting that participant on the spot by not asking them to read or interpret written information for the group. Table 3 presents suggested prompts and their uses.

**Table 3.** Useful design session prompts.

Prompt	Uses
Let’s all move on to the page that looks like this [hold up stimulus].	For in-person sessions; ensures participants are all looking at the same thing.
Talk me through what you think is happening in this [part of the] image.	Elicits interpretation of meaning in a more granular way. May need to add “even if it seems really obvious.”
Can you please tell me more about that?	Encourages participant to elaborate further.
What are you looking at in the image that led you to that conclusion?	Helps to tease out if participant’s comment is based on stimulus or something else, like prior knowledge. Can also help identify graphical elements that may be misleading.
What caught your attention first/stood out to you the most?	Pinpoints the parts of the stimulus that are most salient for the participant. Ideally, these are associated with the most important ideas/content rather than content of lesser importance.
How would you explain this to a loved one in your own words?	Good for visualizations that contain a lot of information because it helps identify the viewer’s main takeaways.
Was any of this information new or surprising to you?	Novel information is often the most important target for comprehension support with visualizations. Helps the session leader ask more focused comprehension assessment prompts.
That is a really interesting idea. Do you think others would see this in the same way?	Validates a participant’s contribution while prompting them to reflect on how widely shared their opinion might be. Useful for comments that one suspects might not generalize well.
Are there any words that we should change to make them more understandable to other people in your community?	Preserves participant dignity by providing socially acceptable cover for raising concerns about difficult words. Participants might respond, “I understand this, but some of our older folks might not.”
What questions do you still have after looking at this information? Is there anything missing?	Identifies gaps in the content.
The next pages all show the same information. Take a look at all of them and then we’re going to vote by show of hands for which you like best.	Re-orients participants to a new task, eg, from elicitation of meaning to expressing preference between options.
I’d like to hear from someone new on this one.	Encourages participation from quieter members of the group, especially if others are dominating the conversation.

### Narration

A skilled leader narrates nonverbal behaviors to facilitate later analysis of the recording. The most important narration identifies the speakers and the part of the stimulus under discussion, such as “David, I see you pointing to the upper left part of the page.” Reactions, such as quiet laughter or pushing the paper away, also merit narration: “Belinda, you’re shaking your head no. Tell me more about what you’re thinking.” It is often possible to follow the participant’s gaze, especially if seated directly across from them, to observe what parts of the page they are looking at [50]. Observation can reveal what areas are the most salient (ie, where participants look first) and can lead to useful prompts (eg, “You looked surprised when you got to the bottom of the page, tell me about that.”).

### Group Dynamics

Group dynamics can influence the productivity of design sessions. For instance, power dynamics can emerge between researchers and participants and therefore must be managed [16,51-53]. Methods to do this include modifying language to be less technical, emphasizing the value of participants’ voices, selecting design activities that participants are comfortable with, and designing from participant-informed stimuli. In addition, design session facilitators will need to guard against 1 or 2 participants dominating the conversation. It can be helpful for the researcher to have prompts prepared to help balance the participation in the session and guide less outspoken participants to provide opinions. Furthermore, factors such as age and gender can influence group interactions and how comfortable participants are speaking freely [54,55]. For instance, we experienced 1 session with older Dominican adults in which the 3 women fell into a pattern of letting or expecting the 1 man to speak first. If they seemed to always defer to his opinion, the leader would have had to gently intervene, but because the pattern was driven by shared cultural expectations and the women still expressed diverse opinions, the leader let it continue. If the participants had had starkly differing expectations about group interaction, the situation would have been trickier to navigate, underscoring the value of grouping participants strategically by important characteristics whenever possible. Group dynamics can also be challenging to manage over video calls. For example, some participants may talk over one another, or their speech might be unintelligible, making some comments unusable by the research team.

### Concurrent Analysis and Debrief

We typically engage in 2 levels of analysis: concurrent and post hoc. Concurrent analysis occurs during and immediately after the design sessions, and its purpose is to support the design decisions that lead to subsequent iterations [56]. Specifically, the goals are to identify pertinent design suggestions, verify the comprehensibility and acceptability of designs, and confirm the completeness of designs (that no important information is missing). Analyses conducted post hoc are discussed in the After Sessions section and are used to uncover broader, potentially generalizable themes.

The focus of concurrent analysis is on how designs are performing in relation to the stated visualization objectives. Therefore, if the objective is comprehension, then the team listens for a match between participants’ interpretations of meaning and the intended meaning. If these are poorly aligned, it is the job of the design session leader to discover why. Further questioning should help identify the source of the problem.

Participants seldom make specific, actionable design suggestions because they are not design experts. However, they can help diagnose problems. Sometimes their diagnoses are general but direct: “This section needs to stand out more.” Other times, they display symptoms that the team must diagnose. For example, if participants are consistently overlooking important information (eg, “Oh, I didn’t notice that”), it might not be prominent enough. It is the researcher’s job to communicate these “diagnoses” to the designer (particularly if the designer is not present during design sessions), so the designer can apply design principles in subsequent iterations (eg, draw attention with a contrasting color) to solve problems.

Research team members present at the session convene immediately afterward for a recorded debriefing to discuss impressions, review notes and key feedback, and come to a consensus about the next steps. The product of concurrent analysis and debriefing is a set of decisions: (1) which stimuli, if any, should be discontinued (we typically show a design in at least 2 sessions before discontinuing), (2) which stimuli should be shown again unchanged, (3) a list of design changes to be made before the next session, (4) design changes under considerations pending due to requirement of further data, and if necessary, (5) any new stimuli that should be created.

## After Sessions

### Transcription and Translation

Transcripts of recordings must be checked for accuracy—preferably by someone who was present at the design session—regardless of whether human or machine transcription was used. High-quality human transcription requires minimal cleaning and is preferable when speakers have heavy accents but takes much longer than machine transcription (d vs min) and is much costlier. Both can be useful within the same project. Even if there are many errors in a machine transcription, its quick availability and automatic time stamps can still be useful for locating specific passages within a recording to support design decisions during concurrent analysis; a more accurate human transcription can be used later for the post hoc analysis.

When key members of our research team are bilingual, we analyze transcripts in their original language, which helps maintain the accuracy and cultural context of the data. Additional time and resources are needed if the transcripts must be translated.

### Post Hoc Analysis

Post hoc analysis is an opportunity to engage more deeply with the data and identify categories and themes that emerged across the entire design process. Multiple inductive approaches to post hoc analysis are possible. We typically use conventional content analysis to examine all study artifacts, including recordings, transcripts, and notes [57]. Post hoc analysis is optional because it does not contribute to the design of the project that generated the data. Rather, the purpose is to identify insights that may be transferrable to other projects in the future. For example, in 1 study with family caregivers of persons with dementia, the participants preferred that infographics related to dementia be scaled such that “good” values are on the left and “bad” values are on the right to match their perception that dementia leads to inevitable decline over time [49]. By contrast, when visualizing the caregiver’s overall health, the same participants preferred “good” values on the right, suggesting a more optimistic frame. This observation led us to conclude that, regardless of the visualization topic, it is important to understand viewers’ frames and scale the visualization to match. In other examples, post hoc analysis has helped us uncover common pitfalls (eg, overly literal interpretation) [26] and preferences for certain kinds of imagery (eg, the subjective experience of symptoms [11]; showing health risks explicitly [58]), among others [10,34,59-61].

### Working Within Resource Constraints

The resources invested in participatory design generally influence the quality of the resulting visualizations. However, participatory design remains a feasible approach even if resources are scarce, as is demonstrated in the study by Stonbraker et al [10], which was completed in the Dominican Republic with a limited budget, and in other projects that have successfully implemented participatory design with limited resources [62-64]. Beyond social considerations, methods to reduce the cost of participatory design while maintaining the integrity of the process include assigning multiple roles to research team members (rather than hiring extra staff), using machine transcription, digitizing materials (eg, projecting prototypes instead of printing them), engaging students in the project, supplementing session data with design surveys, or working with a less-experienced designer to create visualizations. Notably, similar to other researchers [51-53], our team has found that special consideration should be made to minimize the power dynamic that frequently emerges between researchers and participants, as mentioned in the Group Dynamics section. This is especially true in resource-constrained settings when the researchers or other students or professionals from high-income countries are leading or conducting design sessions and participants are members of the local community. In these settings, it is imperative that the researchers or organizations leading the sessions either provide sufficient research staff for the sessions or reach an agreement with local staff on the amount of time they can spend on participatory design. Without this understanding, we have seen research teams lean too heavily on local staff, which creates an uneven burden on those in the local setting. Furthermore, working with the local organization to establish the time and space for participatory design sessions can strengthen partnerships and lead to less stressful and rushed sessions.

## Discussion

### Is It Worth the Trouble?

Participatory design takes time and requires resources and preparation, but we have always found it to be worthwhile, even essential, for producing visualizations that genuinely center the needs and preferences of the intended audience. For example, we have confidently tried out prototype designs that the literature suggested should be successful (eg, Isotype-style infographics [65]) only to have them fail spectacularly [26]. It is very gratifying when designs are successful and participants make comments, such as “Anyone could understand this.” We also find that many participants take pride in their contributions, having done their part to advance visual communication about health in a way they felt would benefit their community. For example, 1 participant said that if she ever saw the infographic in a medical office, she would know that she had made important decisions about how it looked. Consequently, design sessions are worthwhile not only for their creative output but also for their ability to empower and engage participants. Ultimately, the most important result is an impact on target visualization and health outcomes. Although research is still emerging that associates well-designed visualizations with specific health outcomes, such as improved comprehension of intended concepts [12] and understanding of concepts, such as risk [14], better communication [13], and better health behaviors (eg, adherence to medications) [12], this is a growing area, and more research is needed.

### Limitations

Our practical recommendations for conducting participatory design sessions to develop information visualizations are not intended to be exhaustive or definitive; they are constrained by the limits of our experiences. As detailed earlier, there are also various limitations to each approach of participatory design sessions, from the challenges of navigating videoconference calls to the scheduling and transportation limitations of meeting in person. Moreover, successful participatory design research requires organization, planning, engaged interdisciplinary team members, and, importantly, flexibility. We present an overview of challenges and potential solutions but cannot guarantee that other, more nuanced challenges will not occur, particularly outside of the populations we have direct experience working with.

### Conclusions

In this guide, we provide information and recommendations on how to prepare, conduct, and analyze participatory design sessions for information visualizations. We also present the pros and cons of various approaches to participatory design, with the understanding that researchers will select the methods that best match the goals and objectives of their research. We hope that our hard-won lessons learned can streamline and demystify the process for others and thus encourage greater uptake of the method. That said, a written guide can only go so far; all the authors welcome email inquiries and continued discourse via papers and presentations in the spirit of advancing science.

## Supplementary material

10.2196/64508 Multimedia Appendix 1 Expertise and case studies.

10.2196/64508 Multimedia Appendix 2 Informed consent template.

10.2196/64508 Multimedia Appendix 3 Sample design session guide.

10.2196/64508 Multimedia Appendix 4 Design surveys.

10.2196/64508 Multimedia Appendix 5 Design session preparation checklist.

## Abbreviations

ISO: International Organization for Standardization

## References

[1] Botero A, Hyysalo S, Kohtala C (2020). Getting participatory design done: from methods and choices to translation work across constituent domains. Int J Des.

[2] Vandekerckhove P, de Mul M, Bramer WM, de Bont AA (2020). Generative participatory design methodology to develop electronic health interventions: systematic literature review. J Med Internet Res.

[3] Restall G, Diaz F, Faucher P, Roger K (2021). Participatory design and qualitative evaluation of a decision guide for workplace human immunodeficiency virus self-disclosure: the importance of a socio-ecological perspective. Health Expect.

[4] Dindler C, Smith R, Iversen OS (2020). Computational empowerment: participatory design in education. CoDesign.

[5] Alves Villarinho Lima B, Almeida LDA Appropriation for interdisciplinary practice: the case of participatory design in Brazilian computer science.

[6] Khowaja K, Syed WW, Singh M (2022). A participatory design approach to develop visualization of wearable actigraphy data for health care professionals: case study in Qatar. JMIR Hum Factors.

[7] Jänicke S, Kaur P, Kuzmicki P Participatory visualization design as an approach to minimize the gap between research and application.

[8] Ahmed R, Toscos T, Rohani Ghahari R (2019). Visualization of cardiac implantable electronic device data for older adults using participatory design. Appl Clin Inform.

[9] Tiase VL, Wawrzynski SE, Sward KA (2021). Provider preferences for patient-generated health data displays in pediatric asthma: a participatory design approach. Appl Clin Inform.

[10] Stonbraker S, Halpern M, Bakken S, Schnall R (2019). Developing infographics to facilitate HIV-related patient-provider communication in a limited-resource setting. Appl Clin Inform.

[11] Arcia A, George M, Lor M, Mangal S, Bruzzese JM (2019). Design and comprehension testing of tailored asthma control infographics for adults with persistent asthma. Appl Clin Inform.

[12] Stonbraker S, Liu J, Sanabria G (2021). Clinician use of HIV-related infographics during clinic visits in the Dominican Republic is associated with lower viral load and other improvements in health outcomes. AIDS Behav.

[13] Houts PS, Doak CC, Doak LG, Loscalzo MJ (2006). The role of pictures in improving health communication: a review of research on attention, comprehension, recall, and adherence. Pat Educ Couns.

[14] Garcia-Retamero R, Cokely ET (2017). Designing visual aids that promote risk literacy: a systematic review of health research and evidence-based design heuristics. Hum Factors.

[15] Spinuzzi C (2005). The methodology of participatory design. Tech Commun.

[16] Smith F, Wallengren C, Öhlén J (2017). Participatory design in education materials in a health care context. Act Res.

[17] Mackay WE, Beaudouin-Lafon M, Vanderdonckt J, Palanque P, Winckler M (2020). Handbook of Human Computer Interaction.

[18] Schuler D, Namioka A (1993). Participatory Design: Principles and Practices.

[19] Luck R (2003). Dialogue in participatory design. Des Stud.

[20] Galleguillos MLR, Coşkun A (2020). How do I matter? A review of the participatory design practice with less privileged participants.

[21] Stonbraker S, Flynn G, George M (2022). Feasibility and acceptability of using information visualizations to improve HIV-related communication in a limited-resource setting: a short report. AIDS Care.

[22] Semouchkina V (2021). Advancing visual design culture in STEM lab groups [Thesis]. http://hdl.handle.net/1773/47292.

[23] Stalmeijer RE, Mcnaughton N, Van Mook W (2014). Using focus groups in medical education research: AMEE Guide No. 91. Med Teach.

[24] Arcia A, Woollen J, Bakken S (2018). A systematic method for exploring data attributes in preparation for designing tailored infographics of patient reported outcomes. eGEMS.

[25] Mangal S, Berger L, Bruzzese JM (2024). Seeing things the same way: perspectives and lessons learned from research-design collaborations. J Am Med Inform Assoc.

[26] Arcia A, Suero-Tejeda N, Bales ME (2016). Sometimes more is more: iterative participatory design of infographics for engagement of community members with varying levels of health literacy. J Am Med Inform Assoc.

[27] Papoutsi C, Wherton J, Shaw S, Morrison C, Greenhalgh T (2021). Putting the social back into sociotechnical: case studies of co-design in digital health. J Am Med Inform Assoc.

[28] Sanz MF, Acha BV, García MF (2021). Co-design for people-centred care digital solutions: a literature review. Int J Integr Care.

[29] Arcia A, Grossman LV, George M, Turchioe MR, Mangal S, Creber RMM Modifications to the ISO 9186 method for testing comprehension of visualizations: successes and lessons learned.

[30] Arcia A, Velez M, Bakken S (2015). Style guide: an interdisciplinary communication tool to support the process of generating tailored infographics from electronic health data using EnTICE^3^. eGEMS (Wash DC).

[31] Meloncon L, Warner E Data visualizations: a literature review and opportunities for technical and professional communication.

[32] Rajwan YG, Kim GR (2011). Visualization to improve patient learning and communication. SPIE.

[33] Adar E, Lee E (2020). Communicative visualizations as a learning problem. IEEE Trans Visual Comput Graphics.

[34] Arcia A, Spiegel-Gotsch N, George M (2023). Design of tailored asthma control status infographics. Inf Des J.

[35] Becker MH, Radius SM, Rosenstock IM, Drachman RH, Schuberth KC, Teets KC (1978). Compliance with a medical regimen for asthma: a test of the Health Belief Model. Pub Health Rep.

[36] Bandura A (1997). Self-Efficacy: The Exercise of Control.

[37] Glanz K, Bishop DB (2010). The role of behavioral science theory in development and implementation of public health interventions. Annu Rev Public Health.

[38] Klein G, Phillips JK, Rall EL, Peluso DA, Hoffman RR (2007). Expertise out of Context: Proceedings of the Sixth International Conference on Naturalistic Decision Making.

[39] Lakoff G, Johnson M (2008). Metaphors We Live By.

[40] Parsons PC Conceptual metaphor theory as a foundation for communicative visualization design. https://www.viscomm.io/2018/papers/metaphor.pdf.

[41] Eccles MP, Grimshaw JM, Shekelle P, Schünemann HJ, Woolf S (2012). Developing clinical practice guidelines: target audiences, identifying topics for guidelines, guideline group composition and functioning and conflicts of interest. Impl Sci.

[42] Busetto L, Wick W, Gumbinger C (2020). How to use and assess qualitative research methods. Neurol Res Pract.

[43] Morse B, Soares A, Ytell K (2023). Co-design of the transgender health information resource: web-based participatory design. J Particip Med.

[44] DeJonckheere M, Vaughn LM (2019). Semistructured interviewing in primary care research: a balance of relationship and rigour. Fam Med Community Health.

[45] Arcia A, Spiegel-Gotsch N Asthma control infographics, 2017-2018. Columbia Academic Commons.

[46] Stonbraker S, Porras T, Schnall R (2020). Patient preferences for visualization of longitudinal patient-reported outcomes data. J Am Med Inform Assoc.

[47] Airtable.

[48] Fischer CT (2009). Bracketing in qualitative research: conceptual and practical matters. Psychother Res.

[49] Arcia A, Suero-Tejeda N, Spiegel-Gotsch N, Luchsinger JA, Mittelman M, Bakken S (2019). Helping Hispanic family caregivers of persons with dementia “get the picture” about health status through tailored infographics. Gerontol.

[50] Bock SW, Dicke P, Thier P (2008). How precise is gaze following in humans?. Vis Res.

[51] Corsini L, Aranda-jan CB, Cassi H, Moultrie J (2019). Recommendations for participatory design in low-resource settings. Conf Proc Acad Design Innov Manag.

[52] Mejía GM, Guest MA, Zheng W (2024). Who’s ideating, prototyping, and evaluating? A case study of resource-limited participatory design for health and aging. Educ Gerontol.

[53] Harrington C, Erete S, Piper AM (2019). Deconstructing community-based collaborative design: towards more equitable participatory design engagements. Proc ACM Hum Comput Interact.

[54] Curry LA, O’Cathain A, Clark VLP, Aroni R, Fetters M, Berg D (2012). The role of group dynamics in mixed methods health sciences research teams. J Mix Methods Res.

[55] Wheelan SA (2005). The Handbook of Group Research and Practice.

[56] Vindrola-Padros C, Johnson GA (2020). Rapid techniques in qualitative research: a critical review of the literature. Qual Health Res.

[57] Hsieh HF, Shannon SE (2005). Three approaches to qualitative content analysis. Qual Health Res.

[58] Mangal S, Carter E, Arcia A (2022). Developing an educational resource for parents on pediatric catheter-associated urinary tract infection (CAUTI) prevention. Am J Infect Control.

[59] Arcia A, Suero-Tejeda N, Bakken S (2019). Development of pictograms for an interactive web application to help Hispanic caregivers learn about the functional stages of dementia. Stud Health Technol Inform.

[60] Lor M, Yang NB, Backonja U, Bakken S (2023). Evaluating and refining a pain quality information visualization tool with patients and interpreters to facilitate pain assessment in primary care settings. Inform Health Soc Care.

[61] Lor M, Hammes AM, Arcia A (2024). Development of a culturally appropriate faces pain intensity scale for Hmong patients. Pain Med.

[62] Mauka W, Mbotwa C, Moen K (2021). Development of a mobile health application for HIV prevention among at-risk populations in urban settings in East Africa: a participatory design approach. JMIR Form Res.

[63] Asipade AI, Thomas JO, Rankin YA Implementing community-based participatory design and mixed methods to capture and analyze mental models of no/low literate users.

[64] Gregg ES, Colton J, Matin MA, Krupnik TJ (2020). Efficient and participatory design of scale-appropriate agricultural machinery workshops in developing countries: a case study in Bangladesh. Dev Eng.

[65] Haroz S, Kosara R, Franconeri SL ISOTYPE visualization: working memory, performance, and engagement with pictographs.

