# Supplementary material for: A Practical Guide to Participatory Design Sessions for the Development of Information Visualizations: Tutorial
Source: J Particip Med. 2024 Dec 13;16:e64508. doi: 10.2196/64508 (PMC11661693; doi:10.2196/64508)
Supplement: Multimedia Appendix 1 [file jopm-v16-e64508-s001.docx]

**Appendix 1: Expertise and Case Studies**

Dr. Arcia, who began conducting design sessions in 2013, leads our team of nurse scientists. Drs. Stonbraker, Lor, and Mangal are all former trainees of Dr. Arcia who have led their own visualization development studies and made methodological adaptations to meet the needs of their populations and contexts. Our participants have included community members, interpreters, patients, clinicians, and visualization experts across the United States and internationally. We have developed generic and tailored visualizations in English, Spanish, Haitian Creole, Bangla, Chinese, and Hmong for a wide range of topics including pain, HIV self-management, infection prevention, hypertension, asthma, mental health symptoms, dementia caregiving, and COVID-19 in a variety of different settings. See the table below for case studies summarizing how four visualization research studies implemented the recommendations offered in this guide. Consult the referenced articles for complete details on the methods for each study; details of additional participatory design studies by our team can be found in [11, 26].

**Table A1. Case Studies of Visualization Research Studies Using Participatory Design**

|  | Study 1 | Study 2 | Study 3 | Study 4 |
| --- | --- | --- | --- | --- |
|  |  |  |  |  |
| **Name** | New York City Hispanic dementia caregiver Research Program [49] | Information visualizations to facilitate clinician-patient communication in HIV care (Info Viz for Health®)[10,12] | Pain assessment information visualization [60] | Educational resource for parents on pediatric catheter-associated urinary tract infection (CAUTI) prevention [58] |
|  |  |  |  |  |
| **Purpose** | To understand and support the needs of Hispanic family caregivers of persons with dementia | To enhance clinical communication between clinicians and their Latino patients living with HIV | To facilitate communication about pain between Hmong patients with limited English proficiency (LEP), interpreters, and providers during pain assessment | To meet the learning needs of parents about pediatric CAUTI prevention in the hospital setting |
|  |  |  |  |  |
| **Role of visualization** | Return individual research data to participants | Augment patient-provider communication through culturally relevant and visually appealing visualizations. | To reduce the gap in cultural differences in communicating pain information between LEP Hmong patients, interpreters, and providers | Provide educational information to parents using text and visualizations |

|  | Study 1 | Study 2 | Study 3 | Study 4 |
| --- | --- | --- | --- | --- |
|  |  |  |  |  |
| **Visualization topic(s)** | *Self-reported:* caregiving burden, overall health, depression symptoms, psychological distress, self-mastery, care recipient's behavior and cognitive functioning  *Observed:* care recipient’s stage of dementia | Priority information topics to enhance HIV-related self-management such as tips to help with medication adherence, function of CD4 cells, and guides to help with laboratory value interpretations | Pain quality | Topics related to CAUTI prevention: hand hygiene, skin care and assessment, bag care and assessment, warning signs/causes for concern |
|  |  |  |  |  |
| **Location(s)** | New York City | Dominican Republic; New York City, NY; Denver, CO | Wisconsin | Virtual (video conference) in the United States |
|  |  |  |  |  |
| **Variations** | In person groups, audio recorded, elicit meaning, choose best option, feedback, generate new ideas, no stimulus preview | Individual and group sessions, audio recorded, elicit meaning, feedback, generate new ideas, no stimulus preview | Virtual and in-person group sessions, audio recorded, elicit meaning, choose best option, feedback, design surveys, stimulus preview. | Virtual, audio and video-recorded, choose best option feedback, design surveys, no stimulus preview |
|  |  |  |  |  |
| **Design sessions** | 3 English, 3 Spanish; | 10 in-person, group design sessions | 4 group virtual sessions, 4 group in-person sessions with LEP Hmong | 10 English, virtual, individual sessions |
|  |  |  |  |  |
|  | 1 month between first and last session | 8 months in between first and last session | 8 virtual sessions with interpreters | ~2 months between first and last session |
|  |  |  |  |  |
| **Design budget** | $8,000 flat fee for designer based on estimate of 100 hours of work | Design by staff member with relevant expertise (no additional cost) | Approximately $1,000 flat fee for designer based on estimate of hours of work | $4,480 flat fee for designer based on estimated 18 images with iterations. |
|  |  |  |  |  |
|  | Participant incentives $50 x (*N* = 16) | Refreshments for design sessions | Participant incentives $50 x (*N* = 60) | Participant incentives $50 x (*N* = 10); $140 for survey incentives; $50 survey raffle |
|  |  |  |  |  |
|  | Transcription for ~15 hours of audio | Transcription for ~10 hours of audio | Transcription for ~90-100 hours of audio | Transcription for ~8 hours of audio |

**References**

10. Stonbraker S, Halpern M, Bakken S, Schnall R. Developing infographics to facilitate HIV-related patient-provider communication in a limited-resource setting. *Appl Clin Inform*. Aug 2019;10(4):597-609. [doi: [10.1055/s-0039-169400](https://www.thieme-connect.de/products/ejournals/abstract/10.1055/s-0039-1694001)1] [Medline: [31412382](https://pubmed.ncbi.nlm.nih.gov/31412382/)]

11. Arcia A, George M, Lor M, Mangal S, Bruzzese JM. Design and comprehension testing of tailored asthma control infographics for adults with persistent asthma. *Appl Clin Inform*. Aug 2019;10(4):643-654. [doi: [10.1055/s-0039-1693713](http://dx.doi.org/10.1055/s-0039-1693713)] [Medline: [31486056](https://pubmed.ncbi.nlm.nih.gov/31486056/)]

12. Stonbraker S, Liu J, Sanabria G, et al. Clinician use of HIV-related infographics during clinic visits in the Dominican Republic is associated with lower viral load and other improvements in health outcomes. *AIDS Behav*. Dec 2021;25(12):4061-4073. [doi: [10.1007/s10461-021-03331-8](https://link.springer.com/article/10.1007/s10461-021-03331-8)] [Medline: [34129143](https://pubmed.ncbi.nlm.nih.gov/34129143/)]

26. Arcia A, Suero-Tejeda N, Bales ME, et al. Sometimes more is more: iterative participatory design of infographics for engagement of community members with varying levels of health literacy. *J Am Med Inform Assoc*. Jan 2016;23(1):174-183. [doi: [10.1093/jamia/ocv079](https://academic.oup.com/jamia/article/23/1/174/2379938)] [Medline: [26174865](https://pubmed.ncbi.nlm.nih.gov/26174865/)]

49. Arcia A, Suero-Tejeda N, Spiegel-Gotsch N, Luchsinger JA, Mittelman M, Bakken S. Helping Hispanic family caregivers of persons with dementia “get the picture” about health status through tailored infographics. *Gerontol*. Sep 17, 2019;59(5):e479-e489. [doi: [10.1093/geront/gnz085](http://dx.doi.org/10.1093/geront/gnz085)] [Medline: [31185098](https://pubmed.ncbi.nlm.nih.gov/31185098/)]

58. Mangal S, Carter E, Arcia A. Developing an educational resource for parents on pediatric catheter-associated urinary tract infection (CAUTI) prevention. *Am J Infect Control*. Apr 2022;50(4):400-408. [doi: [10.1016/j.ajic.2021.09.006](https://www.ajicjournal.org/article/S0196-6553(21)00593-9/fulltext)] [Medline: [34543706](https://pubmed.ncbi.nlm.nih.gov/34543706/)]

60. Lor M, Yang NB, Backonja U, Bakken S. Evaluating and refining a pain quality information visualization tool with patients and interpreters to facilitate pain assessment in primary care settings. *Inform Health Soc Care*. Oct 2, 2023;48(4):353-369. [doi: [10.1080/17538157.2023.2240411](https://www.tandfonline.com/doi/full/10.1080/17538157.2023.2240411)] [Medline: [37603830](https://pubmed.ncbi.nlm.nih.gov/37603830/)]
