# Supplementary material for: A Practical Guide to Participatory Design Sessions for the Development of Information Visualizations: Tutorial
Source: J Particip Med. 2024 Dec 13;16:e64508. doi: 10.2196/64508 (PMC11661693; doi:10.2196/64508)
Supplement: Multimedia Appendix 2 [file jopm-v16-e64508-s002.docx]

**Appendix 2: Participatory Design Informed Consent Template**

Principal Investigator:

IRB Protocol Title/Number:

Participation Duration:

Anticipated Number of Subjects:

**Research Purpose/Objective:**

We are doing this research study to find out how to make it easier for [intended audience] to understand and use information about their [health condition] through information visualizations like graphs or diagrams. As a first step, we want to figure out if the images that we have designed are [easily understood / persuasive / helping people take X action].

**What information is on this form?**

We are asking you to take part in a research study because you are a [member of intended audience, e.g., an adult with asthma]. This form explains why we are doing this study and what you will be asked to do if you choose to be in this study. It also describes the way we (Researchers) would like to use and share information about you.

Please take the time to read this form. We will talk to you about taking part in this research study. You should ask us any questions you have about this form and about this research study.

You do not have to participate if you don't want to.

**What will I be asked to do if I choose to be in this study?**

This study will last approximately [length e.g., 1-2 hours]. We will ask you to meet as a group and look at a series of images related to [health condition] and ask your opinions about the images. We will analyze what you tell us to help improve the images.

**Audio and Video Recording**

To make sure that no important information is lost, we will [audio and/or video-record] our discussion. Being [audio and/or video recorded] is a requirement of participation; however, data from recordings will only be used to transcribe discussion, and recordings will be stored [secure location] and will be [deleted after X years / deleted after study results are published / kept indefinitely]. The recording(s) will include your name if you choose to use it during the meeting. You can use a fake name on the recording if you choose to.

**Are there any risks?**

The greatest risk of taking part in this study is the possibility of a loss of confidentiality [and, if applicable, any other risks]. Loss of confidentiality includes having your personal information shared with someone who is not on the study team and was not supposed to see or know about your information.

Even though the questions to guide the group discussion are not meant to make anyone uncomfortable or embarrassed, it is possible that some part of the discussion may make you uncomfortable. However, you do not have to answer any question that you do not want to answer, and you can stop talking or leave the group at any time.

**Are there any benefits?**

You may or may not receive direct benefit from taking part in this study. The possible benefit of taking part in this study is that you may learn about [topic area]. Your participation will help to improve the images before they are shown to other people with [health condition].

**What other options are there?**

The alternative is to not participate.

**Confidentiality**

We will make every effort to protect the confidentiality of the design session data, but absolute confidentiality cannot be guaranteed. You will never be named in any material that results from this study. Your information will be assigned a code number, and separated from your name or any other information that could identify you. The research file that links your name to the code number will be kept [description of security measures], and only the investigator and study staff will have access to the file. Other study data, devices, and recordings will be stored in [description of secure locations].

By participating in the session, you grant permission for data to be made available to:

- The investigator and study staff who may be evaluating the study;
- The [Institution Name] Institutional Review Board that independently reviews the study to assure adequate protection of research participants, as required by federal regulations;
- [list any other applicable parties such as the participant’s healthcare provider, the funder, or if the study is federally funded, the Office for Human Research Protections (OHRP) of the Department of Health and Human Services]

**Compensation**

You will receive [compensation, if applicable] for your time.

**Voluntary Participation**

Taking part in this study is your choice. Your choice will not affect the treatment you receive from [affiliated clinical site]. You affirm that have thoroughly read this [information sheet / consent form] and understand the nature and the purpose of the study.

**Contact Information**

If you have any questions about the study, contact [study investigator with phone and email]. If you have any questions about your rights as a research subject, you should contact [institution IRB with phone and email].

**Statement of Consent**

I have read this [information sheet / consent form] and I agree to be in the research study described above. A copy of this [information sheet / consent form] will be provided to me [to keep / after I sign it]. By [agreeing to participate / signing this consent form], I have not given up any of the legal rights that I would have if I were not a participant in the study.
