# Supplementary material for: A Practical Guide to Participatory Design Sessions for the Development of Information Visualizations: Tutorial
Source: J Particip Med. 2024 Dec 13;16:e64508. doi: 10.2196/64508 (PMC11661693; doi:10.2196/64508)
Supplement: Multimedia Appendix 3 [file jopm-v16-e64508-s003.docx]

**Appendix 3: Sample Design Session Guide**

| Good morning/afternoon/evening. Thank you for joining us today. My name is __________ and my role in this project is ________. This is my colleague ____________. She/he/they will be making notes about our conversation. We are studying ways to make it easier for people to understand and use information about ______. This study is funded by __________. | |
| --- | --- |
| *For group sessions:*  Our discussion should last between an hour and a half and two hours. I will be helping to guide the discussion and make sure everybody has a chance to speak but every person does not have to answer every question. As described in the consent form, we will be audio-recording our conversation to make sure that we fully capture the opinions that you express. | *For individual sessions:*  Our discussion should last between an hour and an hour and a half. I will have a series of questions for you but if there’s anything you don’t want to discuss, we can skip it. As described in the consent form, we will be video recording our conversation to make sure that we fully capture your opinions and reactions. |
| We will show you a series of images and ask you a few questions about them. In general, we are interested in knowing   1. what you think we are trying to communicate with the images, 2. which images you prefer and why, 3. how the images can be improved, and 4. what actions you might take as a result of seeing the images.   There are no right or wrong answers to the questions that we will ask. We want your honest opinions. If something is confusing or you don’t like it – that’s exactly the kind of thing we want to know so that we can make it better. We want you to feel comfortable expressing your opinions. To help make that possible, can we agree to keep everything that is said here confidential? I’m going to start the recording now. | |
| *For groups sessions:*  Let’s start off with introducing ourselves. You can use your real name or a fake name, whichever you like. Take a moment to write down the name you would like to use on the name card in front of you and then tell it to the group. As we get started, please remember not to interrupt anyone and try to give everyone a chance to speak. | *For individual sessions:*  For this discussion, you can use your real name or a fake name, whichever you like. What would you like me to call you? |
| *For each image:*   - <Explain the purpose and context, e.g., “your doctor would send this to you by mail”> - What information do you think we are trying to convey with this image? What does it mean to you? - What is your reaction to seeing this information? If this was your information, what would you think or do?   *After small groups of similar images:*   - Which of the images do you prefer? [Use a voice or hand vote for groups] - Why is that your preference? - How can the images be improved?   *Closing*   - Is there anything else we should have asked you?   I would like to remind you that we agreed to keep everything we heard here today confidential. Thanks again for joining us today! | |
