# Supplementary material for: A Practical Guide to Participatory Design Sessions for the Development of Information Visualizations: Tutorial
Source: J Particip Med. 2024 Dec 13;16:e64508. doi: 10.2196/64508 (PMC11661693; doi:10.2196/64508)
Supplement: Multimedia Appendix 4 [file jopm-v16-e64508-s004.docx]

**Appendix 4: Design Surveys**

**Purpose and Item Types**

Some of the tasks typically performed during design sessions can also be accomplished through surveys. For example, we often use the early part of the participatory design process to winnow down the options for the graphical elements that will later be integrated into a larger infographic. We do this by asking participants to choose the best option from among multiple graphical element prototypes. Working on this task synchronously with participants is beneficial because it leaves open the possibility of unexpected insights. However, if the design sessions are done on an individual basis or in small groups, it can take many sessions to gain confidence in having arrived at consensus opinion. By contrast, surveys often are limited in the richness of the data they provide—and as such, should not replace design sessions entirely—but are a good supplement for helping the design team identify consensus quickly. Multiple choice items prompt survey respondents to choose only their most preferred option from among multiple graphical elements. ‘Select all that apply’ items (examples 1 and 2 below) allow respondents to identify all the options they consider acceptable. Optional text fields for open-ended responses can be included to allow respondents to comment on their choices or describe alternatives to the options presented.

***Example 1***

The following statements will be in the brochure. Which image(s)^1^ help get the message across clearly?

“The catheter starts out free of germs, but with time, germs can travel up the tube and cause infection in the bladder.”

| A | B | C | D | | E |  |
| --- | --- | --- | --- | --- | --- | --- |
| 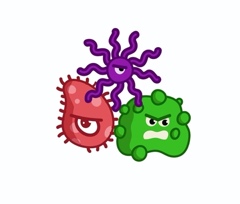 | 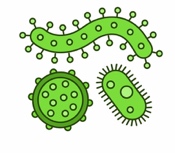 | 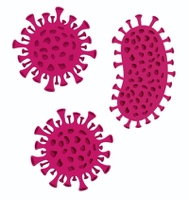 | 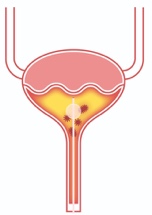 | | 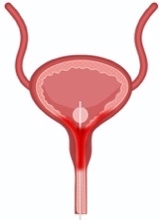 |  |
|  |  |  |  | |  | |
| F | | | | | |  |
| Other (Describe the image that you would prefer): | | | | | |  |
|  | | | |  | |  |

***Example 2***

We are trying to create images that will make messages about COVID-19 clear and easy to understand. For each question in the survey, you will see a description of an idea or message followed by options for images. Please select one or more images that you think shows the idea clearly. If you think they are all clear, pick what you like best. If you have ideas about how to make the images better, you can write them in the box under the images.

A swab that goes to the back of your throat

 A  B  C  None


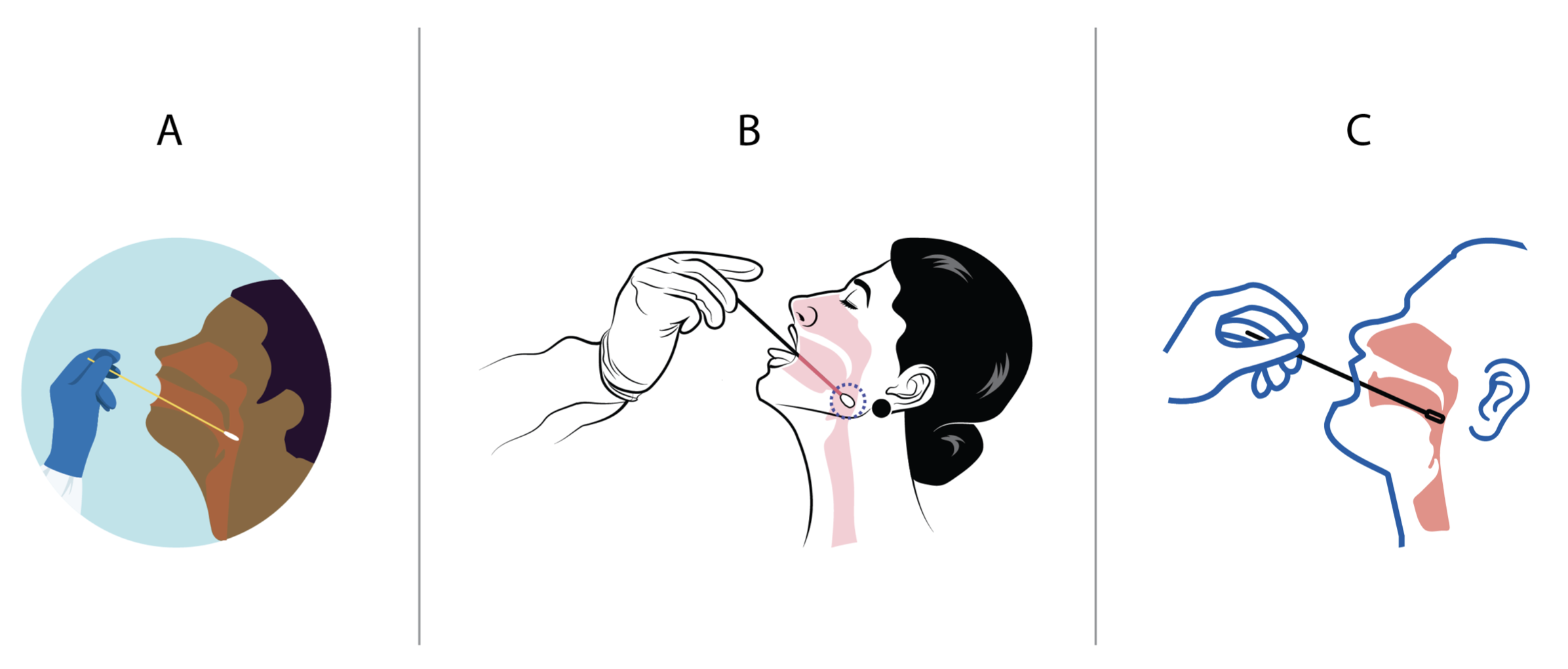


Note that when used at the beginning of the design process, the survey results are just a starting point. In the case of Example 1, design session participants preferred that imagery be even more explicit in conveying pain and infection, as shown in the final design for this graphical element, below.


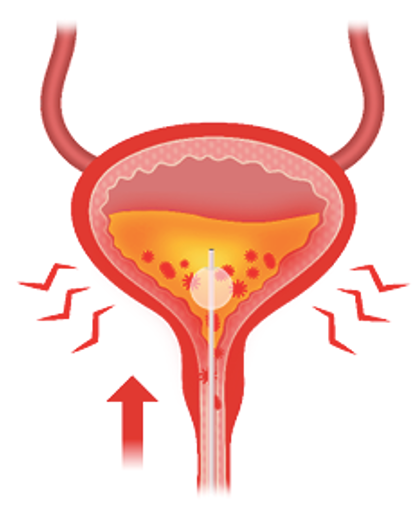


Image of inflamed bladder^2^

In the case of Example 2, A and B were endorsed by roughly equal numbers of survey respondents, so the design team moved forward with a style that was a hybrid of the two and created multiple versions that allow tailoring based on community needs, as shown below.

**
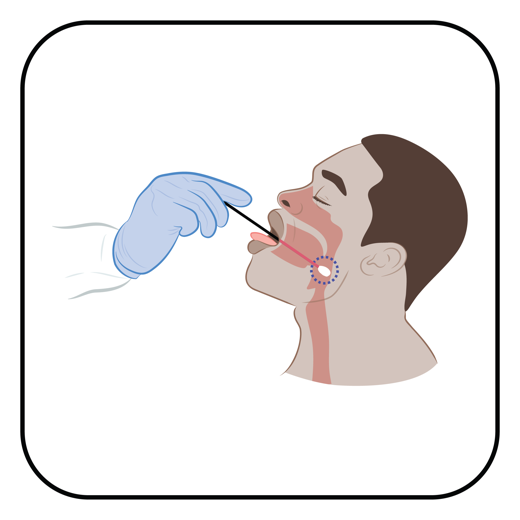
** **
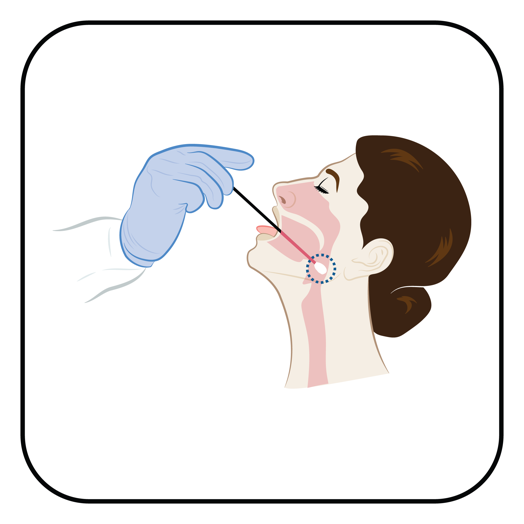
**

Surveys can also be used to understand what informational elements should be prioritized for inclusion or prominence in a visualization. Survey respondents can be asked to rank order the elements in relation to each other or give each informational element an importance rating, as shown in Example 3.

***Example 3***

How important is each of these things to you when learning about washing your hands or using hand sanitizer?

| Show a step-by-step of how to wash your hands/use hand sanitizer correctly. | - Extremely important - Very important - Moderately important - Slightly important - Not at all important |
| --- | --- |

After the completion of participatory design sessions, surveys can be used to help validate the designs including assessing reliability and content validity. For example, in the study conducted with Hmong participants, pictograms were developed to illustrate Hmong cultural metaphors of pain quality descriptions (e.g., “it hurts like a chicken pecking”). Following development, a survey was conducted with a new group of participants including Hmong community members and bilingual Hmong healthcare practitioners to assess if the images could be reliably matched to the cultural metaphors. They were asked to listen to a description of the cultural metaphor and then select the pictogram that best illustrated it.

**Instructions to Respondents**

To give participants sufficient guidance and context to the survey tasks, we suggest providing an introduction segment to discuss the objectives and purpose of the survey. A text introduction can be used, as shown in Example 2. For more complex topics or tasks, an introductory video may be more appealing and accessible for respondents, especially if respondents have limited literacy.

^1,2^ Images © Sabrina Mangal, used with permission.
