# Supplementary material for: A Practical Guide to Participatory Design Sessions for the Development of Information Visualizations: Tutorial
Source: J Particip Med. 2024 Dec 13;16:e64508. doi: 10.2196/64508 (PMC11661693; doi:10.2196/64508)
Supplement: Multimedia Appendix 5 [file jopm-v16-e64508-s005.docx]

**Appendix 5: Design Session Preparation Checklist**

**Consent**

- Specify what type of recording will be used (audio or video), how it will be stored, who will have access to it, and how long it will be retained.
- Specify whether or not agreeing to recording is required for participation in the study.
- Consider asking for permission to contact participants again, either to recruit for other studies or to share study results.

**Participant Instructions**

- Remind participants to bring their glasses or “anything you need to see and read.”
- Remind participants of day and time and ensure they have secured transportation.
- For virtual sessions, ask that whenever possible, they choose a quiet space with minimal distractions. Participants should not be driving during the session.

**Tracking System**

- Implement a system to track every iteration of a stimulus that will be shown to participants. For each stimulus, record:
- a unique identifier
- its relationship, if any, to other iterations (e.g. parent or child)
- reasons for design changes
- when/to whom it was shown — date, time, location, language, number/key characteristics of participants

**Staffing**

- Design session leader
- Note-taker
- Designer/illustrator, if available
- Additional staff as needed, such as a content expert or someone to provide child/elder care in another room

**Supplies, all sessions**

- Notes template with thumbnails and IDs of images. Suggested columns are direct quotes, action items, and observations. Also record date, time, location, language, staff present, participant names/aliases. Draw a seating map.
- Copies of design session guide for staff
- Pens and markers
- Blank paper or notepads
- Digital or analog incentives for participants

**Supplies, in-person sessions**

- Informed consent forms and intake forms (e.g., demographics, health literacy measures, domain-specific instruments)
- Printed copies of all stimuli in the order they will be shown. Ideally, each participant has their own deck and they are watermarked on the back with the unique image ID. Printing on card stock makes the papers easier for participants to handle. If printing multiple decks of images is not feasible, they can also be projected onto a screen or blank wall or presented as a poster.
- Pens and markers and blank paper. Optional: white board or large easel pad for sketching any ideas the participants have or workshopping content
- Recorders and spare batteries. If using a long table, may need two recorders.
- Table cards for names

**Supplies, virtual sessions**

- Video conferencing software with recording ability
- All documents open and prepared for screen sharing
- Print copies of all stimuli for session leader and note-taker to annotate directly
